# Prognostic Value of Plasma and Urine Glycosaminoglycan Scores in Clear Cell Renal Cell Carcinoma

**DOI:** 10.3389/fonc.2016.00253

**Published:** 2016-11-24

**Authors:** Francesco Gatto, Marco Maruzzo, Cristina Magro, Umberto Basso, Jens Nielsen

**Affiliations:** ^1^Department of Biology and Biological Engineering, Chalmers University of Technology, Göteborg, Sweden; ^2^Medical Oncology Unit 1, IOV Istituto Oncologico Veneto (IRCSS), Padova, Italy

**Keywords:** molecular biomarkers, prognostic biomarkers, kidney cancer, systems medicine

## Abstract

**Background:**

The prognosis of metastatic clear cell renal cell carcinoma (ccRCC) vastly improved since the introduction of antiangiogenic-targeted therapy. However, it is still unclear which biological processes underlie ccRCC aggressiveness and affect prognosis. Here, we checked whether a recently discovered systems biomarker based on plasmatic or urinary measurements of glycosaminoglycans (GAGs) aggregated into diagnostic scores correlated with ccRCC prognosis.

**Methods:**

Thirty-one patients with a diagnosis of ccRCC (23 metastatic) were prospectively enrolled, and their urine and plasma biomarker scores were correlated to progression-free survival (PFS) and overall survival (OS) as either a dichotomous (“Low” vs. “High”) or a continuous variable in a multivariate survival analysis.

**Results:**

The survival difference between “High”- vs. “Low”-scored patients was significant in the case of urine scores (2-year PFS rate = 53.3 vs. 100%, *p* = 3 × 10^−4^ and 2-year OS rate = 73.3 vs. 100%, *p* = 0.0078) and in the case of OS for plasma scores (2-year PFS rate = 60 vs. 84%, *p* = 0.0591 and 2-year OS rate = 66.7 vs. 90%, *p* = 0.0206). In multivariate analysis, the urine biomarker score as a continuous variable was an independent predictor of PFS [hazard ratio (HR): 4.62, 95% CI: 1.66–12.83, *p* = 0.003] and OS (HR: 10.13, 95% CI: 1.80–57.04, *p* = 0.009).

**Conclusion:**

This is the first report on an association between plasma or urine GAG scores and the prognosis of ccRCC patients. Prospective trials validating the prognostic and predictive role of this novel systems biomarker are warranted.

## Introduction

Approximately 50% of cases of clear cell renal cell carcinoma (ccRCC), the most common form of kidney cancer (1), develop metastatic disease, which is usually incurable. In sharp contrast to early diagnosed ccRCC, the median survival of metastatic patients is significantly worse (2, 3). The introduction of sequential use of tyrosine-kinase inhibitors (sunitinib, pazopanib, sorafenib, axitinib, lenvatinib, and cabozantinib) and mTOR inhibitors (temsirolimus and everolimus) as well as immunotherapies (interleukin-2 and nivolumab) vastly improved the prognosis of metastatic ccRCC, though with large variation in overall survival (OS) (4–8). These differences highlight the need to identify the critical biological processes underlying ccRCC aggressiveness in order to discover molecular prognostic markers that can subsequently guide the therapeutic choices (9).

To this end, significant advances have been made in the elucidation of the molecular complexity of ccRCC progression (10–13). Using a systems biology approach, we have recently discovered that transcriptional regulation of glycosaminoglycan (GAG) biosynthesis is a prominent event in ccRCC, exacerbated in metastasis (14). Further, we demonstrated that this regulation is mirrored by systemic alterations in subjects’ GAG profile, both in urine and plasma. We designed a plasma and/or urine score that leverages on the GAG profile. These scores reached up to 100% accuracy in the detection of metastatic disease in a case vs. control pilot study conducted on ccRCC subjects. Because of its accuracy and minimal invasiveness, GAG profiling is an attractive novel biomarker for ccRCC and an early example of systems biomarkers.

The primary goal of this observational study was to understand whether the biomarker score correlated with the prognosis of ccRCC patients enrolled in our previous study (14). No pre-specified hypothesis between biomarker scores and survival were made for this exploratory analysis.

## Subjects/Patients and Methods

### Study Design and Patient Selection

This study report was written in compliance with the REMARK guidelines (15). A prospective and consecutive cohort of ccRCC patients had been enrolled in our previous biomarker study at the Instituto Oncologico Veneto, IOV-IRCCS, Padova, Italy. The series was enrolled between January 2013 and June 2015. The patient population considered for the present study included 31 individuals. Inclusion criteria were as follows: a histological diagnosis of ccRCC; any disease stage; patients either receiving systemic treatment for metastatic disease or on follow-up observation without any evidence of disease; and written informed consent. Exclusion criteria were non-clear cell subtypes. Assessment of disease status was based on clinical examination and on computed tomography or other radiological assessments at follow-up. Patients could be receiving different types of oncological treatment at the time of enrollment, but we previously showed that the biomarker score was independent from use or type of drug treatment (14). Patient follow-up period ended on December 2015 and median follow-up time (from day of sampling to event–death or right censoring) was 2.7 years. All patients in this study were examined routinely every 3–6 months during the follow-up period at the same clinic. All deaths were attributed to metastatic cancer. The study was carried out in accordance with the recommendations of the guidelines of the Research Ethics Committee of IOV-IRCCS, Padova, Italy and the participants provided written informed consent in accordance with the Declaration of Helsinki. The present observational study was notified to the Institutional Review Board at IOV-IRCCS, Padova, Italy on January 2013.

### Biomarker Determination

The biomarker score was calculated based on plasma and urine samples taken once in the occasion of a follow-up visit. Whole blood samples were collected in EDTA-coated tubes. The tubes were centrifuged (2,500 *g* for 15 min at 4°C), and the plasma was extracted and collected in a separate tube. Urine samples were collected in polypropilene tubes. All samples were stored at −80°C until they were shipped for analysis in dry ice. GAG measurements were conducted using capillary electrophoresis with laser-induced fluorescence, as previously described (16, 17). Based on these measurements, the plasma and urine biomarkers were scored according to formula derived previously (14) and here reported:

$$\text{Plasma score}=\frac{[6{\text{s CS]}+\text{CS}}_{\text{tot}}}{\frac{3}{10}\frac{\text{[4s CS]}}{[6\text{s CS]}}+[\text{Ns HS]}}$$

$$\text{Urine score}=\frac{\left[\text{Ns6s HS}\right]+60\cdot \text{Charge HS}}{\left[4\text{s CS}\right]},$$

where [6s CS] represents the fraction of the 6-sulfated chondroitin sulfate, [4s CS] represents the fraction of the 4-sulfated chondroitin sulfate, [Ns6s HS] represents the fraction of the N-sulfated 6-sulfated heparan sulfate, [Ns HS] represents the fraction of the N-sulfated heparan sulfate, CS_tot_ is the total concentration of CS (in micrograms per milliliter), and Charge HS is the total fraction of sulfated disaccharides of HS. Of 31 patients enrolled, 30 plasma and 29 urine samples could be successfully scored. Patients with missing scores were omitted from all subsequent analyses.

### Survival Analysis

Survival was calculated as the time between the date of sampling and the time of event. The time of event is defined as right censoring (date of last follow-up without the event) or as date of death in case of OS and date of progression in case of progression-free survival (PFS). Univariate and multivariate survival analyses were performed by fitting a Cox proportional hazard model to estimate the odds-ratio for the variables of interest and the 95% confidence interval. The log-rank statistical test was utilized to determine the significance of the regression. Initial candidate variables were either the plasma score (two missing data) or the urine scores (one missing data), as continuous variables computed as per formula above. For each fluid, the scores were also used to dichotomize patients into two groups, “Low” vs. “High” score, where the median score for that fluid was used as an unbiased cut-off. Kaplan–Meier survival curves were fitted for the two groups, and the statistical significance for survival difference was evaluated using the log-rank test. Two-year survival rates were calculated as the survival probability at the start of the time interval that includes the Kaplan–Meier fit for 24 months. In addition, we performed two additional exploratory survival analyses. In the first case, the analysis was carried out only in the 23 patients with metastatic disease, and, in the second case, the analysis was repeated by calculating survival as the time between the date of start of first-line treatment for metastatic disease (instead of date of sample collection) and the time of event (progression or death or right censoring).

Further variables were considered for regression of survival using a univariate Cox model as above: age (continuous, in years), Easter Cooperative Oncology Group-ECOG performance status (integer, 0–4), Fuhrman grade (categorical, I or II vs. III or IV, four missing data), Heng score (6) (categorical, good vs. intermediate or poor, one missing data), and the neutrophile-to-lymphocyte ratio (continuous, two missing data). Missing data were omitted. A multivariate Cox model was pre-specified using variables reaching statistical significance in the univariate analysis. In addition, we constructed a multivariate Cox model that featured validated prognostic factors: age and performance status. The validity of the proportional hazard assumption was checked using a two-sided *t*-test between transformed survival time and the scaled Schoenfeld residuals. The sample size was not powered specifically for this study, because no prior knowledge on the prognostic value of the plasma/urine scores was available for ccRCC or any related pathology at the time of design of the pilot study (14). We checked for severe overfitting by performing internal validation of the univariate and multivariate models using a bootstrapping algorithm (1,000 bootstraps) and observing the change in Somers’ *D* rank correlation (*D_xy_*) statistics in the original datasets as opposed to the test set. The so-corrected *D_xy_* is reported as a metric for the predictive discrimination of each individual pre-specified model, where *D_xy_* varies between 0 (random discrimination) to 1 (perfect discrimination). Statistical analyses were performed using the packages *survival* and *rms* in R programing language, v. 3.2.3. *p* values <0.05 were considered significant.

## Results

The prospective cohort comprised of 31 patients. Twenty-three patients had metastatic disease and were being treated with sunitinib (*N* = 16), everolimus (*N* = 3), pazopanib (*N* = 2), axitinib (*N* = 1), or were not currently treated (*N* = 1). Eight patients had a former diagnosis of ccRCC with no evidence of metastatic disease at the time of acquisition of blood and urine sample and were thus not treated with antineoplastic drugs. Among the 23 patients with metastatic disease, 14 patients had not been previously treated with other oncological agents, 5 had been previously treated with one line of treatment (of which 1 with sunitinib), and 1 had been treated with multiple lines of treatment (1 missing data). For this cohort, we had previously calculated the plasma and urine scores in 29 (93%) and 30 (97%) patients, respectively (14). The median score was 0.89 (IQR: 0.33–0.96) for plasma and 1.18 (IQR: 0.88–1.49) for urine. For each fluid, we classified patients as either “Low” or “High” depending if the biomarker score was below or above the median score, which was chosen *a priori* as unbiased cut-off. We provide a comparison of standard clinicopathologic features between the two groups in Table 1. None of the patients scored poor according to the Heng model (6).

**Table 1:** **Clinicopathological features in the prospective cohort, in all patients (first column) or stratified according to “Low” and “High” biomarker score in the urine or plasma**.

Factors	All	Stratified upon plasma score		Stratified upon urine score	
	*N* = 31	Low (*N* = 15)	High (*N* = 15)	Low (*N* = 14)	High (*N* = 15)
**Age**	65 (58–77)	67 (61–80)	63 (56–74)	65 (56–74)	65 (58–77)
**Gender**					
Female	9	7	2	6	3
Male	22	8	13	8	12
**BMI**	23.6 (22.5–26.7)	23.2 (23.1–24.5)	26.1 (22.3–28.1)	23.0 (20.3–23.9)	26.1 (23.3–28.0)
**Smoking habits**					
Never smoker	18	9	8	8	9
Ex-smoker	8	1	7	1	6
**Presence of metastasis**					
Yes	23	7	15	6	15
No	8	8	0	8	0
**Tumor stage**					
T1/T1a/T1b	9	7	2	7	2
T2/T2a	10	5	5	3	6
T3>	9	2	6	3	5
N0	16	9	7	8	8
N1	1	0	1	0	1
NX	14	6	6	6	6
**Fuhrman tumor grade**					
Grade 2	14	7	6	5	9
Grade 3	9	4	5	3	5
Grade 4	4	2	2	4	0
**ECOG performance status**					
0	18	10	8	10	7
1	13	5	7	4	8
**Heng classification**					
Good	15	10	5	8	6
Intermediate	15	4	10	5	9
**Neutrophile-to-lymphocite**					
<3	24	10	13	10	13
≥3	5	3	2	2	2
**Biomarker score**	–	0.25 (0.14–0.67)	1.37 (1.10–1.74)	0.75 (0.54–1.10)	1.49 (1.24–1.87)

*Distributions are summarized as median and interquartile ranges in brackets*.

**Figure 1:**
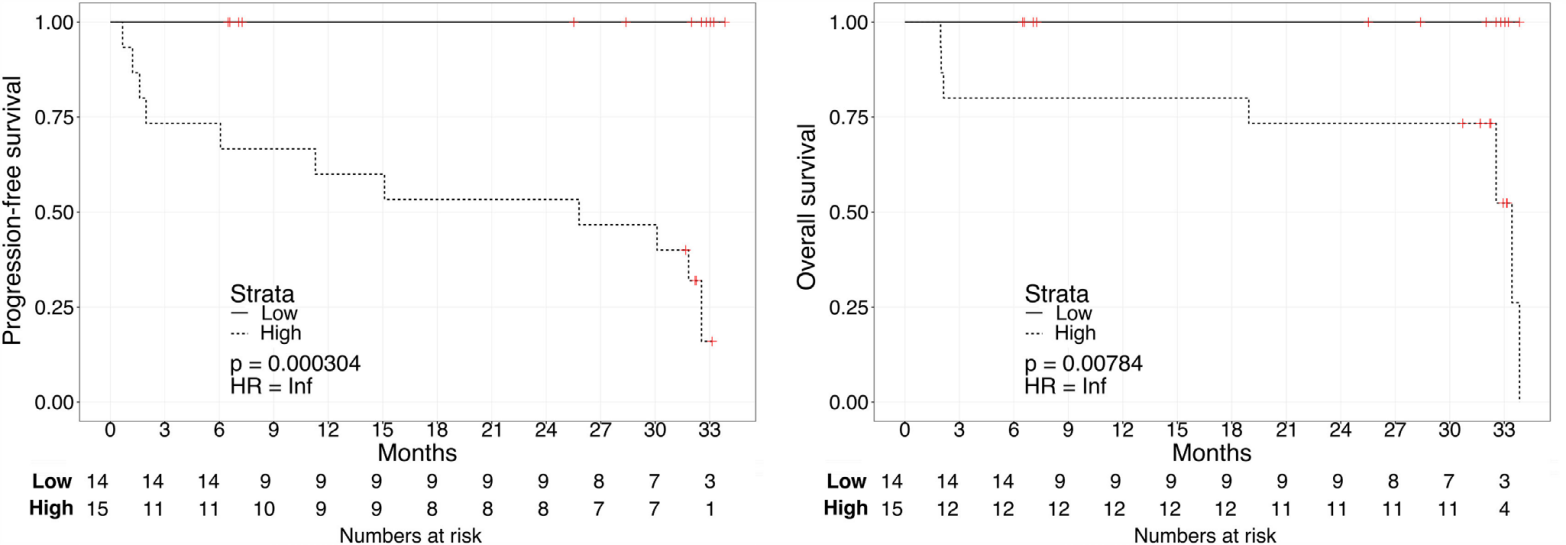
**Kaplan–Meier curves for PFS (left) or OS (right) in ccRCC patients according to urine biomarker score level**. The prospective cohort of patients (*N* = 29) was classified as 14 “Low” (solid) vs. 15 “High” (dashed) biomarker score at the time of sampling.

**Figure 2:**
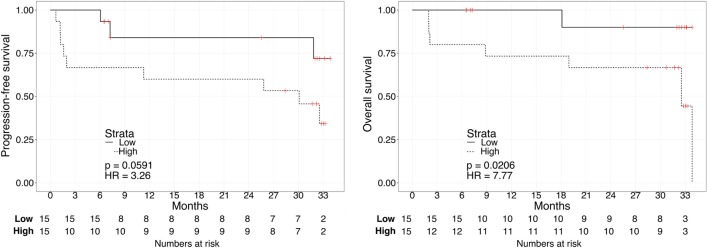
**Kaplan–Meier curves for PFS (left) or OS (right) in ccRCC patients according to plasma biomarker score level**. The prospective cohort of patients (*N* = 30) was classified as 15 “Low” (solid) vs. 15 “High” (dashed) biomarker score at the time of sampling.

Kaplan–Meier survival plots for all 31 patients revealed that “Low”-scored patients fared better both in terms of PFS and OS than “High”-scored patients, both in the case of urine and plasma scores. Notably, despite the limited sample size, the difference between “High” vs. “Low” scores was statistically significant in the case of urine [2-year PFS rate = 53.3 (95% CI: 33.2–85.6%) vs. 100% (Not estimable), log-rank test *p* = 3 × 10^−4^ and 2-year OS rate = 73.3 (54.0–99.5%) vs. 100% (Not estimable), *p* = 0.0078, Figure 1] as well as in the case of OS for plasma [2-year PFS rate = 60 (39.7–90.7%) vs. 84% (66–100%), *p* = 0.0591 and 2-year OS rate = 66.7 (46.6–95.3%) vs. 90% (73.2–100%), *p* = 0.0206, Figure 2]. When modeled as continuous variables, both scores showed a linear and concordant increase in the risk of both PFS and OS, albeit significant only in the case of urine scores [hazard ratio (HR): 10.13, 95% CI: 1.80–57.04, *p* = 0.009 and *D_xy_* = 0.66 for OS; HR: 4.62, 95% CI: 1.66–12.83, *p* = 0.003 and *D_xy_* = 0.57 for PFS]. Estimates for the univariate analysis are reported in Table 2 for PFS and Table 3 for OS.

**Table 2:** **Hazard ratio (HR) for clinical factors and PFS (patients with missing scores were omitted)**.

Factors	*N* (*n* progr.)	HR	Univariate		Multivariate		
			95% CI	*p*-Value	HR	95% CI	*p*-Value
**Age**	29	0.98	0.94–1.03	0.600			
**Fuhrman tumor grade**							
Grade 2	14 (7)	1					
Grade >2	13 (4)	0.39	0.11–1.41	0.393			
**Performance status**							
0	18 (6)	1					
1	13 (7)	2.26	0. 75–6.77	0.146			
**Heng classification**							
Good	11 (6)	1					
Intermediate	14 (7)	0.84	0.28–2.53	0.761			
**Neutrophyle-to-lymphocyte**							
NLR <3	20 (10)	1					
NLR ≥3	4 (3)	2.49	0.68–9.20	0.169	6.92	1.09–44.03	0.040
**Urine biomarker score**	29	4.62	1.66–12.83	0.003	5.38	1.65–17.57	0.005
**Plasma biomarker score**	30	1.69	0.71–4.01	0.232			

**Table 3:** **Hazard ratio (HR) for clinical factors and OS (patients with missing scores were omitted)**.

Factors	*N* (*n* death)	HR	Univariate		Multivariate		
			95% CI	*p*-Value	HR	95% CI	*p*-Value
**Age**	29	0.98	0.94–1.03	0.508			
**Fuhrman tumor grade**							
Grade 2	14 (10)	1					
Grade >2	13 (3)	0.85	0.17–4.26	0.844			
**Performance status**							
0	18 (7)	1					
1	13 (6)	2.06	0.57–7.42	0.268			
**Heng classification**							
Good	11 (3)	1					
Intermediate	14 (7)	2.01	0.52–7.79	0.314			
**Neutrophyle-to-lymphocyte**							
NLR <3	20 (7)	1					
NLR ≥3	4 (3)	4.95	1.15–21.28	0.032	17.77	1.58–200.4	0.020
**Urine biomarker score**	29	10.13	1.80–57.04	0.009	16.43	2.07–130.5	0.008
**Plasma biomarker score**	30	2.23	0.79–6.25	0.127			

**Figure 3:**
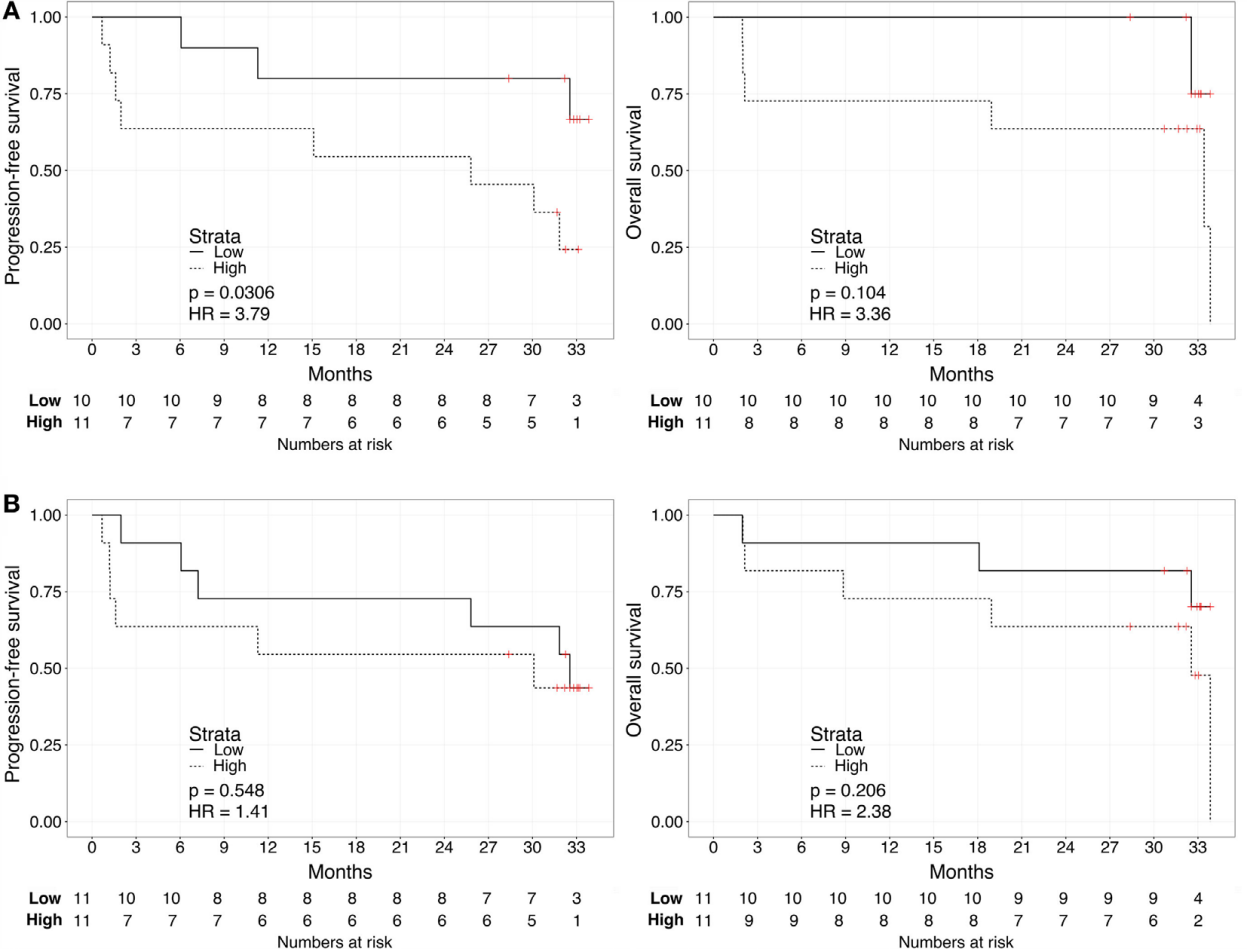
**Kaplan–Meier curves for PFS (left) or OS (right) limited to patients with metastatic ccRCC according to the urine (A) or plasma (B) biomarker score level**. The prospective cohort of patients (*N* = 23) was classified as “Low” (solid) vs. “High” (dashed) biomarker score at the time of sampling.

**Figure 4:**
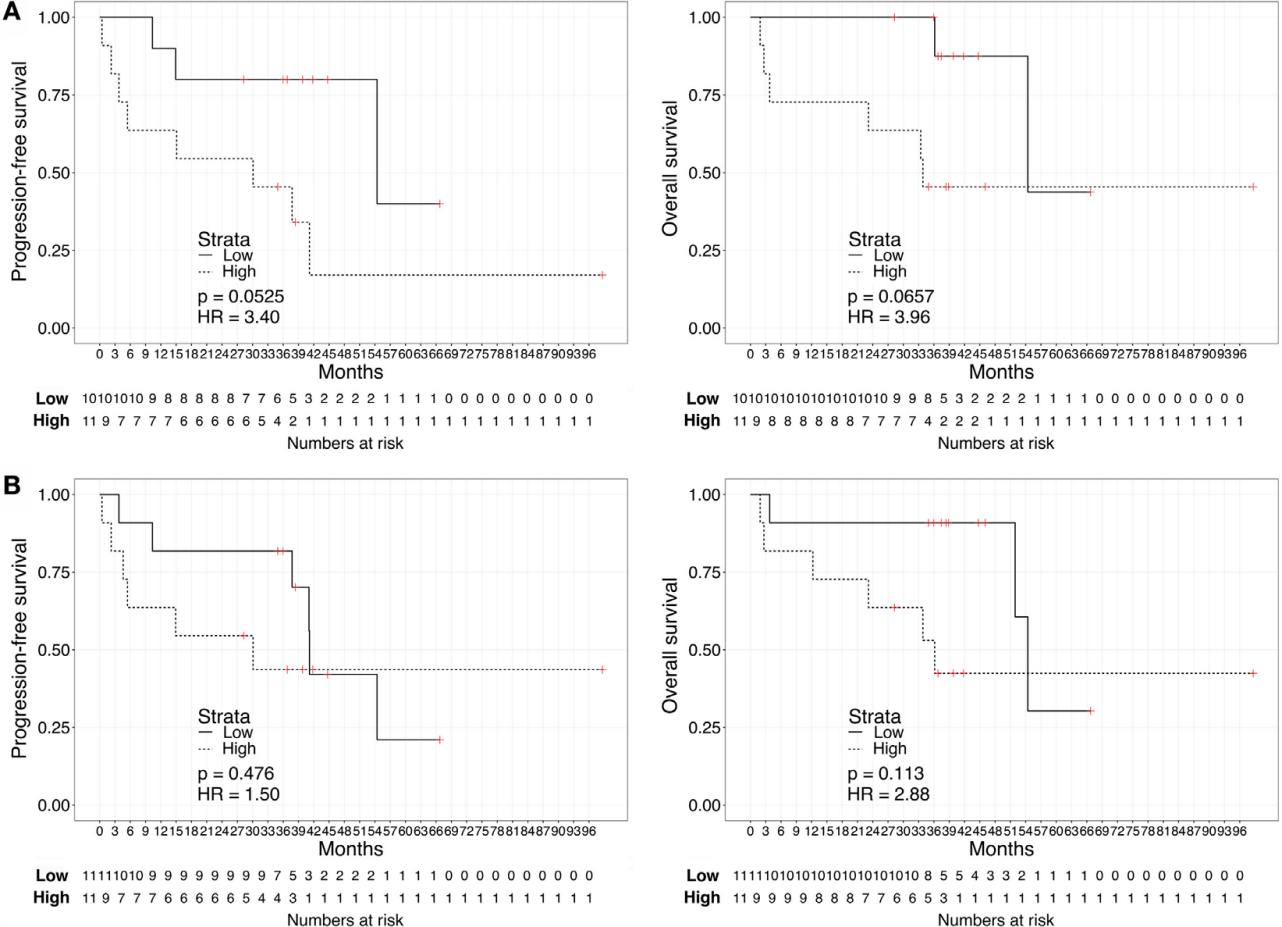
**Kaplan–Meier curves for PFS (left) or OS (right) calculated from date of first treatment start for metastatic disease according to the urine (A) or plasma (B) biomarker score level**. The prospective cohort of patients (*N* = 23) was classified as “Low” (solid) vs. “High” (dashed) biomarker score at the time of sampling.

We repeated the survival analysis above to evaluate two additional scenarios: the correlation between biomarker score and survival in the subset of patients with current metastatic ccRCC diagnosis (excluding eight patients with no evidence of disease); and, within this subset, the correlation between the biomarker score and survival calculated from the start of first systemic therapy. In the first scenario, Kaplan–Meier curves for these patients stratified according to either the urine or the plasma biomarker score underscored a negative association with PFS and OS for “High”-scored patients (Figure 3), although statistically significant only in the case of PFS for urine biomarker [2-year PFS rate = 54.5 (31.8–93.6%) vs. 80% (58.7–100%), *p* = 0.0306]. As continuous variables, we confirmed a statistically significant negative correlation between urine scores and PFS or OS (HR: 3.63, 95% CI: 1.20–10.95, *p* = 0.022 for PFS; HR: 8.40, 95% CI: 1.41–49.96, *p* = 0.019 for OS), while results for the plasma scores were not statistically significant (HR: 0.94, 95% CI: 0.30–2.90, *p* = 0.912 for PFS; HR: 1.64, 95% CI: 0.49–5.45, *p* = 0.420 for OS). In the second scenario, Kaplan–Meier curves showed that “High”-scored patients tended to have worse prognosis in terms of PFS and OS even when survival time was calculated from the date of treatment start (Figure 4). However, the smaller size of this patient subset was underpowered to reach statistical significance in the case of urine scores [2-year PFS rate = 54.5 (31.8–93.6%) vs. 80% (58.7–100%), *p* = 0.0525 and 2-year OS rate = 63.6 (40.7–99.5%) vs. 87.5% (67.3–100%), *p* = 0.0675] as well as plasma scores [2-year PFS rate = 54.5 (31.8–93.6%) vs. 81.8% (61.9–100%), *p* = 0.476 and 2-year OS rate = 63.6 (40.7–99.5%) vs. 90.9% (75.4–100%), *p* = 0.113].

We then evaluated the correlation between survival and other relevant clinical variables of ccRCC: age, performance status, tumor grade, Heng group classification, and the neutrophile-to-lymphocyte ratio (Tables 2 and 3). No significant rank-based internal correlations were observed between these variables and the plasma or urine scores, but age was positively associated with the performance status. We recovered a significant linear increase in the HR for OS (but not PFS) with the neutrophile-to-lymphocyte ratio greater than 3 (HR: 5.03, 95% CI: 1.16–21.80, *p* = 0.031). A multivariate analysis on the plasma or urine biomarker score adjusted for the neutrophile-to-lymphocyte ratio confirmed that the urine biomarker score is an independent predictor of PFS (HR: 4.62, 95% CI: 1.66–12.83, *p* = 0.003) and OS (HR: 10.13, 95% CI: 1.80–57.04, *p* = 0.009), while the plasma biomarker score showed a similar trend without reaching statistical significance (Tables 2 and 3). So-specified multivariate models were also statistically significant and showed remarkable concordance with survival in the case of urine scores (likelihood ratio test *p* = 0.003 and *D_xy_* = 0.63 for OS, *p* = 0.005 and *D_xy_* = 0.47 for PFS), yet not for plasma scores (*p* = 0.053 and *D_xy_* = 0.44 for OS, *p* = 0.224 and *D_xy_* = 0.20 for PFS).

Distinct pre-specified multivariate Cox models that analyzed the estimated effects of the plasma or urine score and established prognostic factors in ccRCC (age and performance status) also provided evidence of statistical associations with survival in the case of urine scores (likelihood ratio test *p* = 0.016 and *D_xy_* = 0.50 for OS, *p* = 0.021 and *D_xy_* = 0.48 for PFS). However, the individual coefficients for the biomarker scores did not reach statistical significance in these models, neither for urine (HR for OS: 4.79, 95% CI: 0.71–32.09, *p* = 0.107 and HR for PFS: 3.01, 95% CI: 0.97–9.35, *p* = 0.057) nor for plasma (HR for OS: 1.69, 95% CI: 0.54–5.31, *p* = 0.362 and HR for PFS: 1.42, 95% CI: 0.51–3.93, *p* = 0.497).

## Conclusion

While metastatic ccRCC is considered invariably incurable, patients may reach widely different survival rates according to clinical prognostic factors (6). In addition, rare complete responses have been reported with current antiangiogenic oncological targeted therapies, with or without metastasectomy (18). Therefore, it is crucial to determine which biological processes underlie the aggressiveness of ccRCC progression, as these could differentiate patients at higher risk and advocate distinct strategies of treatment. In the recent years, several molecular prognostic factors have been shown to effectively predict poor prognosis based on altered expression of proteins or small molecules (19–21). However, these biomarkers typically comprise one or few molecules and are, hence, unlikely to capture the complexity of the key biological processes driving ccRCC aggressiveness. On the contrary, these processes emerge from the network of interactions of several biomolecules (22).

In our recent report, we adopted an innovative systems biology approach to identify the importance of GAG biosynthesis regulation in ccRCC. We discovered that the simultaneous measurements of key GAGs in the plasma and urine effectively capture the regulation of this process, and validated the diagnostic value of scores of this novel systems biomarker (14). This systems biomarker agglomerates measurements at the metabolite level, which represents an alternative layer of biological information with respect to genetic, protein, or immunological markers, which have been extensively investigated as potential novel biomarkers for ccRCC (23–25).

In the present study, we aimed to explore the correlation of the biomarker scores with PFS and OS in the prospective cohort of ccRCC patients enrolled in our previous study. A key limitation of this analysis is the small sample size of our cohort, which could not be powered for survival analysis during the design of our previous study because no data on similar prognostic biomarkers were available in the public domain. We aimed to minimize overfitting by internal validation of the pre-specified multivariate Cox models, yet only larger study populations will provide more precise estimates of HRs for the biomarker scores. Another limitation is that potential technical variabilities in the analytical measurements of GAGs were not addressed here; in that, these measurements were performed in a single laboratory. Finally, our previous study did not find significant correlations between the GAG scores and dietary or lifestyle habits (14), but this relation could not be further controlled in the present analyses, due to limited data points. For analogous reasons, future studies should focus on variation on GAG scores attributable to removal of primary tumor (nephrectomy or enucleoresection) or to the time of appearance of distant metastases, concomitantly or subsequently after diagnosis of ccRCC.

Despite these limitations, the strength of the association between biomarker scores and survival was so high to reach statistical significance in both plasma and urine when patients were grouped depending on the median score. As a continuous variable, the urine score achieved the strongest correlation with poor survival, and in particular for OS (univariate HR = 4.62 for PFS and 10.13 for OS), even when limited to the sole metastatic patients (univariate HR = 3.63 for PFS and 8.40 for OS). In addition, the urine score was independently associated with OS and PFS in the multivariate analysis (multivariate HR = 5.38 for PFS and 16.43 for OS). The plasma score, on the other hand, displayed a weaker trend, which was stronger in the case of OS (univariate HR = 1.69 for PFS and 2.23 for OS). Consistent with the weaker association, this score, as a continuous variable, never reached statistical significance in our cohort. We conjecture that the plasma score could still have some prognostic value because patients with extreme scores fared worse than patients with low scores, as demonstrated by the results when subjects were dichotomized based on the median plasma score (log-rank HR = 3.26 for PFS and 7.75 for OS). Taken together, these results constitute first time evidence that both the plasma and urine biomarker scores at the time of sampling could predict prognosis of ccRCC patients, both in terms of OS and PFS, and that there exists a quantitative linear increase of the risk with increasing scores. These findings demonstrate that our previously discovered GAG-based systems biomarker has not only diagnostic potential, but may also have also prognostic role.

Current prognostic factors in ccRCC are predominantly based on clinical parameters. These include composite scoring systems designed to improve the prognostic value of individual factors, such as tumor size or grade (26). The Heng group classification adopted in our study is an example of such systems (6). An inherent disadvantage of these systems is that prognosis is based on risk groups rather than quantitative prognostic variables. Nevertheless, no prognostic model based on biomarkers has yet been integrated in the routine clinical practice. Limited to blood-based biomarkers, promising results were shown in connection with serum VEGF levels (27) (HR = 1.19 for PFS and 1.39 for OS), serum amyloid A (28) (HR = 2.51–2.81 for OS), and serum insulin-like growth factor-1 (29) (HR = 0.62 for OS). It was noted that these biomarkers could suffer from a number of confounding factors that have no tumor origin (26). For example, VEGF levels may be derived from damaged platelets, while amyloid A is a renowned marker of trauma and various inflammations. Conversely, we and other groups previously provided evidence that the increase of GAG levels in ccRCC patients is likely a product of the tumor itself (30), possibly due to the upregulation of the GAG biosynthetic pathway (14). In addition, the here-reported HRs for the urine score were not only predictors of poor survival in a continuous an independent fashion, but also of much higher magnitude compared to the above mentioned biomarkers (multivariate HR = 5.38 for PFS and 16.43 for OS). However, these values might be inflated by the relatively small sample size, as shown by the wide range of the confidence intervals.

The distribution of GAGs scores appeared to be independent from other prognostic factors such as age, performance status, tumor grade, Heng group classification, and the neutrophile-to-lymphocyte ratio. Since all 8 non-metastatic ccRCC patients had low GAG scores (consistent with the notion that the biomarker correlates with disease severity), we performed an additional analysis in which only the 23 patients with metastatic disease were considered. Once again, GAG score retained its prognostic significance, suggesting that the prognostic role of this biomarker is likely not an expression of the presence of metastatic disease by itself. Finally, even though the samples were taken at different times during patient follow-up, the positive correlation with OS measured since date of first systemic treatment for advanced disease allow us to hypothesize that the prognostic role of the systems biomarkers is independent from the time point of assessment.

These biomarkers have several potential advantages, the first being the minimal invasiveness of measurements, which enables dynamic monitoring of the disease. The biological significance of GAGs might be related to the role of these macromolecules in the interactions with the extracellular matrix and the activation of chemokines (31, 32), both processes being implicated in tumor metastasis. For example, altered expression of GAGs was associated to the differential invasive phenotype between non-invasive brain lesions and glioblastoma multiforme (33). We speculate that a similar mechanism could apply also to ccRCC; even though mechanistic studies are currently missing.

In conclusion, this is the first report correlating the different profiles of plasma and urine GAGs with PFS and OS of patients with ccRCC. The results of this exploratory study are too preliminary to warrant the clinical utility of the GAG score as a prognostic biomarker for ccRCC patients, but provide strong rationale to conduct prospective confirmatory clinical studies. Overall, capturing the complex expression of GAGs by means of a non-invasive systems biomarker opens an exciting field in the quest to develop prognostic biomarkers for ccRCC.

## Author Contributions

FG performed statistical analyses. UB, CM, and MM coordinated the blood and urine sampling and collected clinical data. FG and JN conceived and designed the study. FG wrote the manuscript. All the authors edited and approved the manuscript in its final form.

## Conflict of Interest Statement

FG and JN are listed as inventors in a patent application related to the biomarker described in this study. UB, CM, and MM declare no conflict of interest.
